# Development of Bullous Disease during Treatment of Pulmonary Marginal Zone B-Cell Lymphoma

**DOI:** 10.1155/2012/146081

**Published:** 2012-08-30

**Authors:** S. Ansari, H. Dubaybo, E. Levi, B. A. Dubaybo

**Affiliations:** ^1^Department of Internal Medicine, Wayne State University School of Medicine, 4201 St. Antoine, UHC-2E, Detroit, MI 48201, USA; ^2^Medical Service, John D. Dingell VAMC, Detroit, MI 48201, USA

## Abstract

We describe an unusual case of severe pulmonary bullous disease developing during treatment of marginal zone B-Cell lymphoma (MALT) involving the pulmonary parenchyma. The patient originally presented with pneumonia-like symptoms along with hemoptysis and was diagnosed with MALT lymphoma after a video-assisted thoracic surgical (VATS) lung biopsy. Computed tomography (CT) of the chest at diagnosis revealed multiple opacities, but no bullous disease. During the ensuing 4 years, and while on chemotherapy for the MALT lymphoma, sequential CT and pulmonary function tests revealed the development of progressive bullous disease resulting in the replacement of large portions of the lung parenchyma with bilateral bullae. This complication is rare, has been reported only once before in a patient with concomitant amyloidosis, and may be related to activation of proteolytic enzymes by lymphoma cells or chemotherapeutic agents.

## 1. Introduction

Marginal zone B-cell lymphoma (MALT) is a form of extranodal non-Hodgkin's lymphoma reported in several organs including thymus [1], salivary glands [2], stomach [3], and small intestines [4]. Pulmonary involvement is rare and may manifest as solitary or multiple lesions, diffuse parenchymal infiltrates, and masses or consolidations [5]. Associated findings include air bronchograms, airway dilatation, thoracic lymph node enlargement, and pleural effusions [5]. Pulmonary MALT is indolent, responds to therapy with good prognosis, and has not been associated with chronic pulmonary disability. Specifically, bullous disease has not been reported to accompany its progression or treatment. MALT cells, however, produce proteases [6] that can potentially result in emphysematous lung injury and formation of bullae. This case demonstrates the development of bullous disease in a patient with pulmonary MALT. 

## 2. Case Report

### 2.1. Clinical Presentation

**Figure 1:**
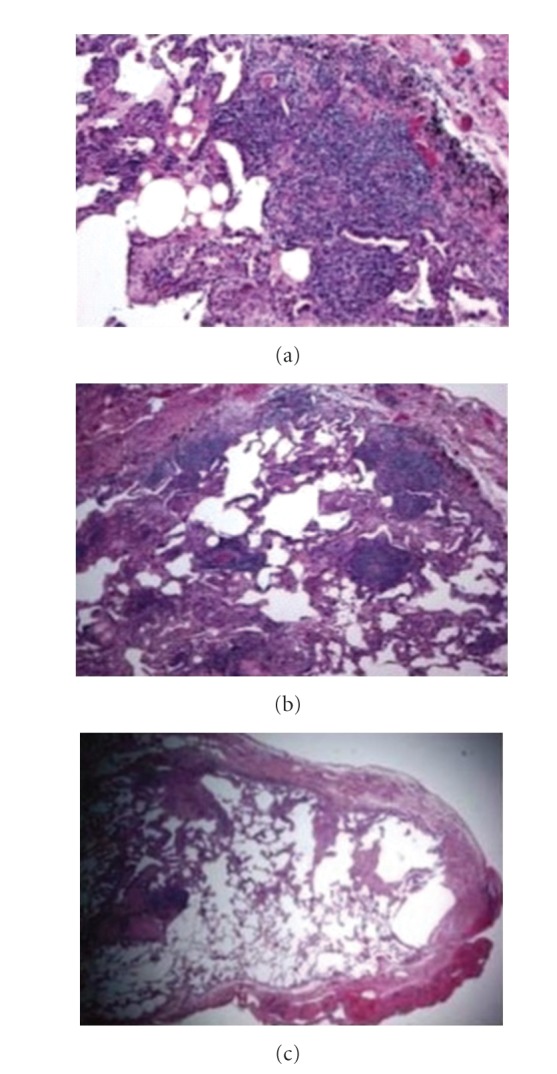
(a) Sections of intraparenchymal lymph node revealing complete effacement of normal nodular architecture by monotonous proliferation of small monocytoid lymphoid cells typical of marginal zone B-cell lymphoma. (b) Lung biopsy showing morphologically pulmonary lymphoid infiltrates with small lymphocytes distributed along the interlobular septae, visceral pleura and vessels, consistent with pulmonary parenchymal involvement by lymphoma. (c) Section of the pulmonary parenchyma showing normal alveolar architecture except in areas close to lymphocytic infiltration where microscopic bullae are seen.

This 70-year-old male first presented in 1991 at the age of 49 for persistent cough. Detailed smoking history revealed that he had been smoking one pack a day since 1971 but had quit a few weeks before his presentation because of persistent cough. Clinical examination and laboratory studies did not show evidence of airway obstruction, gastroesophageal reflux, postnasal drip, or respiratory infection. Chest radiographs were reported to show left lower lung field opacity (Chest radiographs obtained in 1992, 1993, and 1997 films were archived and could not be reviewed by the authors. Information in the text is from radiology reports.) which remained unchanged on multiple films in 1992 and 1993. Cough subsided and this opacity was thought to be clinically insignificant. In 1997, during a routine visit, chest radiographs were repeated and showed, along with the left lower lobe opacity, right lower lobe opacity. Since the patient was asymptomatic, it was decided to follow him clinically. In 2001, the patient developed symptoms of pneumonia, and a computed chest tomography (CT) revealed nonspecific opacities in all lobes suggestive of bilateral consolidative process (The films from this CT were archived and could not be reviewed by the authors). No bullae were described. The patient also developed thrombocytopenia and was diagnosed with idiopathic thrombocytopenia purpura (ITP). His pulmonary symptoms were controlled with antibiotics and bronchodilators. The patient was discharged on bronchodilators, Danazol and systemic corticosteroids for the diagnoses of COPD and ITP. From 2001 to 2007 the patient had multiple episodes of fever, cough and dyspnea which were treated as pneumonias or COPD exacerbations, and he became oxygen dependent. In 2007, he presented with hemoptysis. Bronchoscopy with a BAL and a transbronchial biopsy showed mature lymphocytes. A lung biopsy through a video assisted thoracic surgery (VATS) procedure was performed and the results showed MALT lymphoma (Figure 1). 

### 2.2. Lung Biopsy Findings

Sections of intraparenchymal lymph nodes revealed complete effacement of normal nodular architecture by monotonous proliferation of small monocytoid lymphoid cells (Figure 1(a)). On immunohistochemical studies, these cells were positive for CD20 and bcl-2, but negative for CD5, CD23, CD10, CD43, bcl-6, and bcl-1. No light chain restriction was detected for kappa and lambda light chain. CD3 was reactive with the background lymphocytes. Molecular studies IgH rearrangement and t(11;18) were positive. The lung parenchyma showed morphologically similar lymphoid infiltrate distributed along the interlobular septae, visceral pleura, and vessels, consistent with pulmonary lymphoma (Figure 1(b)). Special stains for amyloid were negative. The light microscopic morphology, immunophenotype, and molecular studies were typical of MALT. Microscopic bullae were detected in areas adjacent to lymphomatous pulmonary infiltrates (Figure 1(c)). 

### 2.3. Clinical Progress

**Figure 2:**
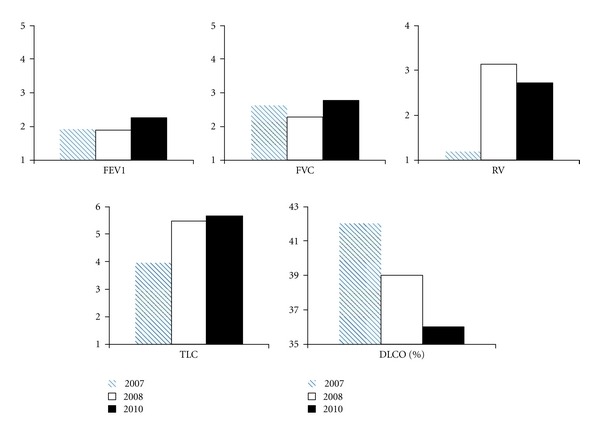
Serial pulmonary function tests from 2007 to 2010 show stable airflow parameters with gradual increase in total lung capacity and residual volume indicating air trapping. Diffusing capacity also progressively decreased consistent with a reduction in gas exchange units as seen in bullous emphysema. Forced expiratory volume at 1 sec (FEV1), forced vital capacity (FVC), total lung capacity (TLC), and residual volume (RV) are expressed in liters (L). Diffusing capacity (DLCO) is expressed as percent (%) of predicted.

The patient was started on chlorambucil for MALT and maintained on danazol, and predniosone for ITP and bronchodilators for presumed COPD. Between 2007 and 2010, the patient had multiple admissions for cough, and fever, which were treated with antibiotics and bronchodilators, with partial improvement. He remained oxygen dependent, continued to use bronchodilators, and needed albuterol several times a day. The pulmonary function tests (PFTs) in 2007, 2008, and 2010 showed stable airflow, progressive decline in diffusing capacity, and progressive air-trapping (Figure 2). Alpha 1 antitrypsin levels were normal.

### 2.4. Chest Computed Tomography Findings

**Figure 3:**
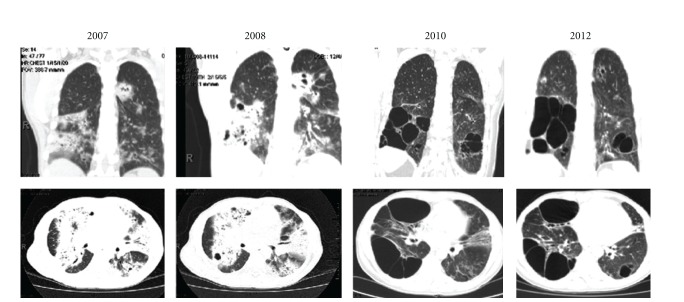
Serial chest computed tomograms showing progressive replacement of areas of lymphomatous interstitial infiltrates with bullous disease.

 Multiple thoracic CT scans were performed to evaluate symptoms and monitor lymphoma progression (Figure 3). No bullous disease had been described in the CT of 2001, and none could be seen in the CT of 2007. In 2008, right lower lung fields showed early formation of bullae which became extensive and bilateral in the CT performed in 2010 and 2012. 

## 3. Discussion

Our patient presented with chronic symptoms of cough, sputum production, fever, and dyspnea. Due to his 20 pack-year history of tobacco smoking, these symptoms were initially attributed to COPD exacerbations and pneumonia. It was not until he had hemoptysis that lung tissue biopsy was obtained resulting in the diagnosis of MALT lymphoma.

Pulmonary bullous disease is generally linked to smoking [7] where bullae typically occur in upper lobes. Less common causes include alpha-1-antitrypsin deficiency where bullae have a predisposition for lower lobes [8], sarcoidosis [9], and pulmonary amyloidosis [10, 11], where bullae occur in areas of lung parenchymal involvement. Bullous disease is not described in the setting of MALT lymphoma. Literature review identified one case of bullous disease in a patient with pulmonary amyloidosis who developed MALT lymphoma [11]. In that case, the bullous disease was attributed to the pulmonary amyloidosis. Our patient had multiple chest radiographs and CT's between 1991 and 2007 which did not show pre-existing bullae. Since making the diagnosis of pulmonary MALT lymphoma and initiation of chemotherapy in 2007, CT scans demonstrated progressive and extensive bullous disease. It is noteworthy that special stains for amyloid in lung biopsy sections were negative.

The mechanism responsible for the development of bullae in our patient is not certain. There may be several likely explanations. MALT cells produce proteolytic enzymes with the potential to induce lung injury and emphysema [6]. The production of these enzymes may have altered the delicate balance between proteolytic and antiproteolytic enzymes in this patient with 20 pack-years of smoking. Alternatively, pathogenesis may be related to inflammatory ball and valve like function of nodular lesions in the airway caused by the MALT lymphoma. MALT lymphomas characteristically involve the airways with intraluminal lesions which occasionally create a ball-valve effect [6, 12]. As MALT tumors are indolent and chronic, the ball-valve effect over years may result in distal parenchymal destruction and necrosis [13]. 

Could the development of destructive lung disease be related to agents used to treat MALT lymphoma and ITP? Chlorambucil can cause pneumonitis and has been linked to the development of pulmonary fibrosis [14–16] while danazol has been reported to be associated with the development of pulmonary fibrosis [17]. To our knowledge, neither agent has been reported to induce bullous disease. In fact, both agents appear to have protective effects on lung parenchyma and activate processes that inhibit intrinsic proteases. Danazol increases the production of antiproteolytic enzymes [18], particularly in patients with alpha 1 antitrypsin deficiency [19] while chlorambucil inhibits conversion of pro-enzymes to active proteolytic agents [20]. It is not known what effect the combination of danazol and chlorambucil might have on lung injury. However, since each appears to favor antiproteolysis, it is unlikely that the combination would promote proteolysis.

Since the patient had normal alpha 1 antitrypsin levels, a congenital or acquired deficiency cannot account for the bullous transformation. Similarly, smoking is unlikely to have contributed to this transformation. The patient had stopped smoking seventeen years before there was evidence of any bullous disease, and serial PFT's did not show any prior to the diagnosis of MALT. We believe inflammatory mediators produced by lymphoma cells and released at an accelerated rate during lymphoma treatment have contributed to parenchymal bullous transformation.
